# Hereditary combined deficiency of the vitamin K-dependent clotting factors

**DOI:** 10.1186/1750-1172-5-21

**Published:** 2010-07-14

**Authors:** Mariasanta Napolitano, Guglielmo Mariani, Mario Lapecorella

**Affiliations:** 1 Haemophilia and Thrombosis Centre, University of L'Aquila, Italy

## Abstract

Hereditary combined vitamin K-dependent clotting factors deficiency (VKCFD) is a rare congenital bleeding disorder resulting from variably decreased levels of coagulation factors II, VII, IX and X as well as natural anticoagulants protein C, protein S and protein Z. The spectrum of bleeding symptoms ranges from mild to severe with onset in the neonatal period in severe cases. The bleeding symptoms are often life-threatening, occur both spontaneously and in a surgical setting, and usually involve the skin and mucosae. A range of non-haemostatic symptoms are often present, including developmental and skeletal anomalies. VKCFD is an autosomal recessive disorder caused by mutations in the genes of either *gamma-glutamyl carboxylase *or *vitamin K2,3-epoxide reductase complex*. These two proteins are necessary for gamma-carboxylation, a post-synthetic modification that allows coagulation proteins to display their proper function. The developmental and skeletal anomalies seen in VKCFD are the result of defective gamma-carboxylation of a number of non-haemostatic proteins. Diagnostic differentiation from other conditions, both congenital and acquired, is mandatory and genotype analysis is needed to confirm the defect. Vitamin K administration is the mainstay of therapy in VKCFD, with plasma supplementation during surgery or severe bleeding episodes. In addition, prothrombin complex concentrates and combination therapy with recombinant activated FVII and vitamin K supplementation may constitute alternative treatment options. The overall prognosis is good and with the availability of several effective therapeutic options, VKCFD has only a small impact on the quality of life of affected patients.

## Disease name and classification

Hereditary combined vitamin K-dependent clotting factors deficiency (VKCFD) is a rare inherited coagulation defect that forms part of a wider group of rare disorders named Familial Multiple Coagulation Factor Deficiencies (FMCFDs). FMCFDs are characterized by the simultaneous decrease in the levels of two or more coagulation factors. The first classification of FMCFDs was proposed by Soff and Levin in 1981 [1,2]. Recently a new classification has been outlined (Table 1) and we currently define FMCFDs as the *presence of more than one coagulation factor deficiency arising from a genetic defect or defects and transmissible as a familial trait *[3]. The development of this new classification required both laboratory and genetic recognition of patients affected by concomitant independent defects as well as the identification of multiple defects related to one single gene mutation. Three subgroups of disorders are therefore included: i) FMCFDs arising from single coagulation factor deficiencies ii) FMCFDs arising from a single genetic defect iii) FMCFDs arising from cytogenetic abnormalities [3]. Since VKCFD arises from a single genetic defect of either *γ-glutamyl carboxylase *(*GGCX*) or *vitamin K 2,3-epoxide reductase complex *(*VKORC*) - two proteins of the vitamin K cycle - in the revised classification it obviously falls in the second subgroup.

**Table 1:** updated classification of the FMCFDs*

***FMCFDs arising from co-incidental single coagulation factor deficiencies:***

• Combined VWD and FXI deficiency

• Combined VWD and haemophilia A

• Combined VWD and haemophilia B

• Combined haemophilia A and FXI deficiency

• Other rarer co-incidental disorders



***FMCFDs arising from single genetic defects:***



*FMCFDs with bleeding as the dominant clinical feature:*

• Combined FV and FVIII deficiency

• Combined vitamin K-dependent coagulation factor deficiency



*FMCFDs with bleeding as part of a wider syndrome complex:*

• Congenital disorders of glycosylation

• Noonan syndrome

• Inborn errors of liver synthetic function or bile secretion



***FMCFDs arising from cytogenetic defects:***

• 13q34 deletion syndromes (combined FVII and FX deficiency)

* Familial Multiple Coagulation Factor Deficiencies

## Definition

VKCFD is a heterogeneous coagulation disorder consisting of a deficiency of clotting factors II (FII), VII (FVII), IX (FIX), X (FX), as well as the coagulation inhibitors protein C (PC), protein S (PS) and protein Z (PZ). The disease leads to a bleeding tendency with a variegate clinical picture. Two subtypes have been identified, deriving from mutations of two enzymes of the vitamin K cycle: VKCFD type1 is defined by defective GGCX activity, first reported in Devon Rex cats [4], while VKCFD type 2 derives from functional deficiency of VKORC [5].

## Epidemiolgy

VKCFD is a very rare autosomal recessive disorder, with a low incidence (< 30 kindreds worldwide). As the disease is inherited in an autosomal recessive manner, the male to female ratio is 1:1. The first case of VKCFD was described in 1966 in an infant girl who presented bleeding from the first days of life [6]. The proband had low or undetectable plasma levels of coagulation factors II, VII, IX and X in the absence of any hepatic disease or malabsorption. Only a few additional cases of VKCFD have been reported to date [7-15]. The scientific literature in this area is composed of single case reports and small clinical series described in Africa, Asia, Europe and North America. Racial distribution, ethnic predilection and carrier incidence are therefore unpredictable.

## Clinical description

Clinical symptoms of VKCFD vary according to procoagulant protein levels which depend on the availability of vitamin K. The severity of the bleeding pattern is therefore influenced by both dietary intake of vitamin K and functional status of the gut microflora, as well as by the penetrance of the genetic defect which is widely variable. Accordingly, in the most severe cases, onset of symptoms occurs in newborns while a delayed recognition of the disorder is possible in milder cases. Despite a modest propensity to thrombosis hypothesized in milder cases due to the deficiency of natural anticoagulants, VKCFD is characterized by a cluster of different, often life threatening, bleeding symptoms occurring both spontaneously and in a surgical setting. Reviewing the scientific literature on this disease, the spectrum of bleeding symptoms appears to range from mild to severe and usually involves skin and mucosae. Easy bruising is common. Muco-cutaneous bleeding, such as gastrointestinal bleeding, may also appear spontaneously or after antibiotic therapy, because of the decreased vitamin K production by gut bacteria. Bleeding from the umbilical cord is reported [6,8]. Hemarthrosis is rarely described [8]. VKCFD can sometimes cause fatal intracranial haemorrhage in the first weeks of life, which is similar to the haemorrhagic disease the newborns that results form acquired vitamin K deficiency [6,7,16]. The case of a woman with persistent menorrhagia, but whose worst bleeding episodes occurred mainly in a surgical scenario, such as post-partum haemorrhage and haemoperitoneum following ovarian cyst rupture, has been described [17]. Antibiotic and anticonvulsivant therapy administration must be carefully evaluated as these drugs can worsen the bleeding pattern [8].

## Non-haemostatic manifestations

VKCFD-affected patients often show a variegate pattern of non-haemostatic symptoms due to defective γ-carboxylation of proteins other than the clotting factors. Developmental and skeletal abnormalities resembling those seen in warfarin embryopathy are striking non-haemostatic features, consisting in stippling of the long bones epiphyses and shortness of finger distal phalanges [7,11]. Osteoporosis without the classical serum circulating markers of bone rearrangement [11,17] and pseudoxanthoma elasticum-like disorders [18] have been reported. As a consequence of the bone impairment during embryogenesis, an increased rate of foetal loss has been suggested by some authors. The exact incidence is still difficult to calculate because of the rarity of the defect [11,19].

## Etiopathogenesis

**Figure 1:**
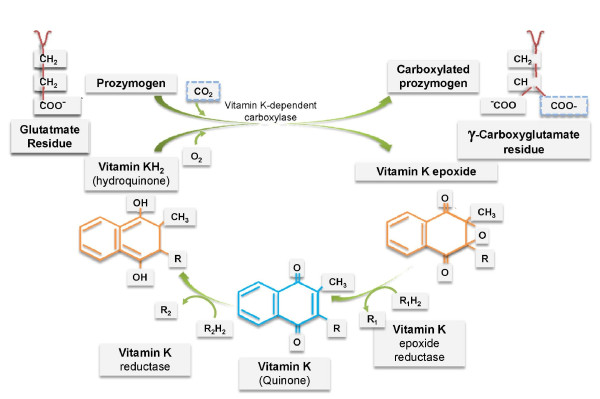
**the vitamin K cycle**.

As with all other coagulation factors, FII, FVII, FIX, FX and the three anticoagulant proteins (PC, PS and PZ) are normally synthesized in the liver. All such factors undergo a post-translational modification of glutamate (*Glu*) residues into γ-carboxyglutamate (*Gla*) residues. Carboxylation, necessary for their normal activity in the coagulation system, involves *Glu *residues located in a homologous '*Gla *domain' which spans approximately 45 amino acids. *Gla *residues enable these proteins to adapt to calcium-dependent conformation allowing their binding to phospholipids [20]. The γ-carboxylation is held in the endoplasmic reticulum and is catalyzed by the enzyme GGCX which is hypothesized to bind to the propeptide coagulation factors at their amino terminus. Carboxylated proteins are then transported to the Golgi for secretion and the propeptide sequence is then removed [21,22]. Vitamin K is an essential cofactor for GGCX and when a carbon dioxide is added to *Glu *to form *Gla*, the reduced form of vitamin K (vitamin K hydroquinone) is oxygenated to form vitamin K 2,3 epoxide. At this stage the enzyme VKORC is needed to regenerate the vitamin K hydroquinone, completing the so called vitamin K cycle [21] (Figure 1). Three forms of vitamin K are known: i)vitamin K1 (Phylloquinone) which is abundant in green and leafy vegetables and is produced by plants and algae; ii)vitamin K2 (Menaquinones) which is a mixture of molecules produced by the microbial intestinal flora and differs from K1 by unsaturated side chains of isoprenoid units with different length; iii)vitamin K3 (Menadione) which is a synthetic form and is more water-soluble.

In VKCFD abnormal carboxylase function arises from defects in the genes encoding either the *GGCX *(VKCFD type 1) or the *VKORC *(VKCFD type 2). In both cases the disorder leads to under-carboxylation and comparable reduction in the activities of several proteins including vitamin K-dependent clotting factors. GGCX was isolated in 1991 by forward genetics and to date several point mutations have been detected [23,24]. One exception is a 14 bp deletion in intron 1, which is supposed to be involved in the protein expression [13]. VKCFD type 1 has been shown to result from compound heterozygosity of missense mutations in the *GGCX *gene [19,25-27]. VKOR was identified 34 years ago [28] but its biochemistry has been difficult to understand because of refractoriness to purification. Both isolation and characterization of VKOR were performed only in 2004 by reverse genetics and expression cloning [29]. The wider knowledge of this protein has amplified the interest of the scientific community in this rare disease. Only one mutation has been recognized to be causative of VKCFD in three different kindreds of Lebanese, German and Italian origin: a single 292C > T nucleotide substitution in the *VKOR complex subunit 1 *(*VKORC1*) that causes the amino acid change Arg98Trp [30,31] in a position close to the vitamin K binding site. Remarkably, mutations other than the Arg98Trp influence the response to vitamin K intake during oral anticoagulation, encoding for resistance to warfarin treatment [32]. VKCDF is therefore a natural model that resembles oral anticoagulation with vitamin K antagonists and treatment of this disease can be used to test different modalities to reverse warfarin overdose, whose most feared and dramatic complication is intracranial haemorrhage.

GGCX and VKORC are also involved in the post-translational processing of non-haemostatic proteins. These include: osteocalcin, PROX and matrix-Gla protein (MGP) expressed in the connective tissue [33]; Gas 6, a protein related to protein S but involved in the process of phagocytosis of apoptotic cells [34,35]; nephrocalcin A-D [36]; several putative carboxylated proteins identified through nucleotide database screening such as proline rich *Gla *protein 1 (PRGP1) and 2 (PRGP2), transmembrane *Gla *protein 3 (TMG3) and 4 (TMG4) [37]. Most non-haemostatic manifestations that complete the clinical picture in individuals affected by VKCFD are explained by defective synthesis of these proteins. A list of haemostatic and non-haemostatic proteins requiring γ-carboxylation to display proper function is reported in table 2.

**Table 2:** haemostatic and non-haemostatic proteins requiring γ -carboxylation to display proper function

Coagulation Factor	MW (KDa)
Factor II	72

Factor VII	50

Factor IX	55

Factor X	58.9

Protein C	62

Protein S	69

Protein Z	68

Bone Proteins	

Osteocalcin	58

Matrix Gla protein	10

Others	

Gas 6 Protein	75

Nephrocalcin	14

PRGP1	23

PRGP2	17

TIMG3	25.8

TIMG4	25.4

The last remarkable pathogenetic aspect of this rare coagulation defect is the role that under-carboxylated factors (PIVKA) may play as signal messengers. In particular, it has been demonstrated that Des-γ-carboxyprothrombin (PIVKA-II), besides being a prognostic marker of hepatocellular carcinoma (HCC), can bind to a receptor for hepatocyte growth factor named *Met*, and has a role as an autologous growth factor for HCC via activating the well known Jak/STAT signalling pathway [38-40]. As vitamin K is the mainstay of treatment of VKCFD, a disease with circulating PIVKA, understanding the kinetics of γ-carboxylation will help to explore the role of this compound as an adjuvant in HCC treatment, which is currently under investigation.

## Diagnosis and differential diagnosis

Diagnosis of VKCFD requires anamnestic differentiation from acquired forms of the disorder attributable to intestinal malabsorption of vitamin K in the case of inflammatory bowel diseases or celiac disease, severe liver dysfunction during liver cirrhosis or accidental ingestion of warfarins and superwarfarins. Such conditions must be excluded with certainty before raising the suspect of the disease. A vitamin K assay with a normal result would be useful for screening them but it is usually only performed in selected research laboratories only. In addition, VKCFD must be distinguished by other congenital clotting defects. These include: single coagulation defects such as haemophilia B (FIX deficiency); isolated deficiencies of FVII, which is considered the most frequent among rare coagulation disorders; the rarest FII and FX deficiencies, as well as combined defects like combined FVII and FX deficiency. The latter is the result of chromosomal abnormalities (chr13q deletions) as the genes encoding for the two factors are located very close in the chromosome 13. Among acquired bleeding disorders, a distinction from the presence of circulating antibodies with inhibitory function such as acquired haemophilia or the very rare presence of inhibitors against FVII is mandatory. VKCFD usually becomes evident because of an excessive bleeding pattern compared to the decreased levels of each of the clotting factors involved, but clinical distinction is not always easy and requires precise laboratory assessment as well as genotype confirmation.

Laboratory assessment is characterized by prolonged prothrombin time (PT) and activated partial thromboplastin time (aPTT). The extent to which the PT and aPTT are prolonged may be influenced by the degree to which each individual coagulation factor is decreased. FII, FVII, FIX and FX activity levels can show variably reduced values. The first clotting factor usually assayed is FVII, because it has the shortest half life, with the PT slightly more prolonged than the aPTT. Abnormal PT and aPTT shorten after mixing with normal plasma. PC, PS and PZ activities are also reduced but, as mentioned, an increased thrombotic risk in VKCFD has only been anecdotally reported [13]. Once seriously suspected, genotyping of *VKORC *(spanning 5 kb and containing 3 exons on chromosome 16) [29] and *GGCX *(spanning 13 kb and containing 15 exons on chromosome 2) [41] can be performed in selected research laboratories. This test is mandatory in order to exactly define a complex coagulation defect that must be distinguished from many acquired conditions as well as from other single or multiple congenital coagulation defects.

## Antenatal diagnosis

There is no report in the scientific literature for antenatal diagnosis in VKCFD. Considering that the disease can be easily treated with vitamin K, the advantages deriving from the procedure are nonessential compared to the potential bleeding complications. Administration of vitamin K in the third trimester of pregnancy in women suspected to carry a child with VKCFD would avoid major haemorrhages in the newborn.

## Treatment

Vitamin K administration is the mainstay of therapy in VKCFD. Nevertheless response to treatment is extremely variable depending both on the administration route and on individual sensitivity to vitamin K [20]. Mildly affected patients can be treated with oral vitamin K which usually only partially corrects the clotting times as restoration of carboxylated coagulation factors is incomplete. However such treatment prevents major haemorrhages. Administration of an oral dose of 10 mg twice or three times per week usually avoids frequent muco-cutaneous bleeding. Alternatively, if not tolerated perorally, the same dose of vitamin K can be regularly administered intravenously at intervals which can vary according to the prothrombin time - international normalized ratio (PT-INR) values. It is important to remark that, despite the generally acknowledged efficacy of vitamin K, a fixed therapeutic schedule does not exist for this rare disease. Additional information on the kinetics of vitamin K-dependent clotting factors come from two recent studies in which the modifications of coagulation phenotype after an intra-venous 10 mg single dose of vitamin K have been tested in VKCFD type2. Such a dose proved to be clinically beneficial and able to provide valuable patterns of factor specific biosynthesis, half-life and decay [17,31]. Unlike other authors have reported [42], restoration of the four coagulation factors was virtually complete with amelioration of the clotting times starting at the 4-hour post-infusion assay. Normalization of the PT-INR was obtained at the 24 -hour post-infusion assay. The effect was still present compared to the patient's basal PT-INR at the 72-hour post-infusion assay. Of the two natural anticoagulants, PC behaved similarly to the other vitamin K-dependent coagulation proteins, while PS levels where only partially restored. Reasons for such differences need further investigation but as a whole, these studies indicate that the fast improvement of the clotting times is due to the rapid release of the coagulation factors accumulated in the uncarboxylated form and undergoing γ-carboxylation, while the sustained normalization results from γ-carboxylation of proteins of '*de novo*' synthesis. Differences in the synthetic pattern may exist for each factor. Furthermore, other reductating pathways, which work with less intensity under physiologic conditions, may contribute to reconstitute the reduced form of vitamin K when saturated by external administration [43,44].

Treatment challenges remain regarding the management of both major surgery and the most severe bleeding episodes. Such occurrences need to be managed in a specific environment such as haemophilia centres. Insights in this field would improve knowledge about warfarin reversal to which this rare disease resembles both clinically and biologically. This is one of the most debated issues among blood coagulation specialists and most information about how to treat VKCFD derive from warfarin reversal.

Plasma supplementation during surgery or overt haemorrhage is indicated. The recommended dose is 15-20 mL/Kg of body weight given intravenously, to be repeated until clinical efficacy and amelioration of clotting times. The requirement of multiple administrations to reach efficient clotting may be complicated by circulatory overload [45].

Prothrombin Complex Concentrates (PCCs) are a series of products containing factors II, VII, IX and X as well as PC and PS at variable concentrations. All PCCs contain factors II, IX and X: those with negligible amount of FVII are commonly known as 3-factor PCCs while, if the amount of FVII is substantial, the product is labelled 4-factor PCC. Both 3 and 4-factor PCCs could also be successfully administered in VKCFD considering their efficacy in reversing warfarin anticoagulation [46]. Specific experience in VKCFD is unreported and therapeutic schedules are therefore derived from the treatment of warfarin reversal, with a suggested dose of 500 U given intravenously (median 8.8 U/Kg) for a PT-INR below 5 [46]. The risk of transmission of blood-born pathogens, although theoretically possible, is extremely low due to viral inactivating procedures, while a low but significant thrombotic risk must be taken into account [47].

Recombinant activated FVII (Eptacog alfa (activated)) (rFVIIa, Novoseven^® ^Novo Nordisk, Copenhagen, Denmark) is a powerful drug with a short half-life (< 4 h) belonging to the so called "by-passing agents". It is indicated for the treatment of haemophilia complicated by the presence of anti-factor VIII (FVIII) inhibiting antibodies which impair the use of FVIII concentrates. Additional indications are congenital FVII deficiency and Glanzmann's disease but rFVIIa has been successfully used in a number of off-label bleeding situations including warfarin overdose or critical bleeding during warfarin treatment within the normal range [48]. It was successfully used in VKCFD during an emergency minor surgical setting [17]. A total dose of 1.2 mg (~ 20 μgr/Kg of body weight) provided a sudden normalization of the PT-INR with the shortest value at the 4-hour post-infusion assay as expected because of the increased FVII. Interestingly, a double rate increase in FII, FIX and FX activities was detected, remarking the role of rFVIIa as a powerful activator of the whole coagulation system, a finding that only a complex defect with multiple factors deficiency allowed the opportunity to explore. Since rFVIIa and vitamin K show different peaks of action (4 hours and 24 hours respectively), their combined use with a simultaneous infusion at the above mentioned doses can obtain a sustained normalization of clotting times to be used in case of major bleeding and more complex surgical procedures. This "combination therapy" is a feasible alternative option for life-threatening bleeding episodes in VKCFD.

## Prognosis

All the mentioned therapeutic options make VKCFD a disease with good prognosis and a low impact on the quality of life, with permanent disabling consequences only resulting in severely affected patients who have been diagnosed after critical events such as intracranial haemorrhage.

## Unresolved questions and future research directions

VKCFD is a very rare disease, but its peculiar pathogenetic mechanism emphasizes its importance far beyond an audience of coagulation specialists. Warfarin is a drug taken by millions of patients worldwide but still has some bleeding complications feared by most practitioners. Sensitivity and resistance to warfarin depend basically on single nucletide polymorphisms of both *VKORC *and *Cytochrome p450 2C9 *genes. Understanding the biological steps from synthesis to secretion of coagulation factors make VKCFD a tool to add additional information in warfarin dose finding and dose limiting studies. Advances in the treatment of VKCFD would help to minimize bleeding complications occurring during oral anticoagulation with vitamin K antagonists by administering a combination of drugs with different mechanisms and peaks of action.

Defective carboxylation of non-haemostatic proteins is still an open issue in some widespread diseases: post-menopausal women show higher circulating levels of uncarboxylated osteocalcin compared to pre-menopausal women [49]. Furthermore, in patients with inflammatory bowel diseases, the malabsorption of fats including liposoluble vitamins results in skeletal impairment. Increasing knowledge in this field discloses a possible role for vitamin K supply.

As a last important research field, the regulation of the vitamin K cycle and the signalling driven by under-carboxylated clotting factors in HCC opens new horizons for vitamin K as an adjuvant in cancer therapy.

## Abbreviations

aPTT: activated partial thromboplastin time; FII: factor II; FVII: factor VII; FVIII: factor VIII; FIX: factor IX; FX: factor X; FMCFDs: Familial Multiple Coagulation Factor Deficiencies; GGCX: γ-glutammyl carboxylase; HCC: hepatocellular carcinoma; MGP: matrix-Gla protein; PC: protein C; PCCs: Prothrombin Complex Concentrates; PIVKA: undercarboxylated vitamin K-dependent clotting factors; PRGP1: proline rich *Gla *protein 1; PRGP2: proline rich *Gla *protein 2; PS: protein S; PT-INR: prothrombin time - international normalized ratio; PZ: protein Z; rFVIIa: recombinant activated factor VII; TMG3: transmembrane *Gla *protein 3; TMG4: transmembrane *Gla *protein 4; VKCFD: vitamin K-dependent clotting factors deficiency; VKORC [1]: vitamin K2,3-epoxide reductase complex (subunit 1)

## Competing interests

The authors declare that they have no competing interests.

## Authors' contributions

All three authors were personally involved in some of the studies mentioned in the references section. MN and ML extensively reviewed the literature about the diseased and wrote the paper. GM reviewed the draft. All authors read and approved the final version of the manuscript.
